# An atlas to support the progressive control of tsetse-transmitted animal trypanosomosis in Burkina Faso

**DOI:** 10.1186/s13071-021-05131-4

**Published:** 2022-03-04

**Authors:** Lassane Percoma, Jean Baptiste Rayaissé, Geoffrey Gimonneau, Zakaria Bengaly, Sié Hermann Pooda, Soumaïla Pagabeleguem, Rasmané Ganaba, Adama Sow, Rafael Argilés, Jérémy Bouyer, Moussa Ouedraogo, Weining Zhao, Massimo Paone, Issa Sidibé, Ouedraogo/Sanon Gisele, Giuliano Cecchi

**Affiliations:** 1 Insectarium de Bobo-Dioulasso-Campagne Panafricaine d’Eradication de la Tsé-tsé et de la Trypanosomose, Bobo-Dioulasso, Burkina Faso; 2 Ecole de Lutte Anti-Tsétsé, Bobo-Dioulasso, Burkina Faso; 3 Centre International de Recherche—Développement sur l’Elevage en zone subhumide, Bobo-Dioulasso, Burkina Faso; 4 CIRAD, UMR INTERTRYP, Montpellier, France; 5 INTERTRYP, Univ Montpellier, CIRAD, IRD, Montpellier, France; 6 Université de Dédougou (UDDG), BP 176, Dédougou, Burkina Faso; 7 Agence de Formation, de Recherche et d’Expertise en Santé pour l’Afrique (AFRICSanté), 01 BP 298, Bobo-Dioulasso 01, Burkina Faso; 8 Food and Agriculture Organization of the United Nations, Emergency Centre for Transboundary Animal Diseases (ECTAD), Conakry, Guinea; 9 Joint FAO/IAEA Centre of Nuclear Techniques in Food and Agriculture, Vienna, Austria; 10 CIRAD, UMR, ASTRE, Montpellier, France; 11 Food and Agriculture Organization of the United Nations, Animal Production and Health Division, Rome, Italy

**Keywords:** *Glossina*, Tsetse, Database, African animal trypanosomosis, Map, GIS

## Abstract

**Background:**

African animal trypanosomosis (AAT), transmitted by tsetse flies, is arguably the main disease constraint to integrated crop-livestock agriculture in sub-Saharan Africa, and African heads of state and governments adopted a resolution to rid the continent of this scourge. In order to sustainably reduce or eliminate the burden of AAT, a progressive and evidence-based approach is needed, which must hinge on harmonized, spatially explicit information on the occurrence of AAT and its vectors.

**Methods:**

A digital repository was assembled, containing tsetse and AAT data collected in Burkina Faso between 1990 and 2019. Data were collected either in the framework of control activities or for research purposes. Data were systematically verified, harmonized, georeferenced and integrated into a database (PostgreSQL). Entomological data on tsetse were mapped at the level of individual monitoring traps. When this was not possible, mapping was done at the level of site or location. Epidemiological data on AAT were mapped at the level of location or village.

**Results:**

Entomological data showed the presence of four tsetse species in Burkina Faso. *Glossina tachinoides*, present from the eastern to the western part of the country, was the most widespread and abundant species (56.35% of the catches). *Glossina palpalis gambiensis* was the second most abundant species (35.56%), and it was mainly found in the west. *Glossina morsitans submorsitans* was found at lower densities (6.51%), with a patchy distribution in the southern parts of the country. A single cluster of *G. medicorum* was detected (less than 0.25%), located in the south-west. Unidentified tsetse flies accounted for 1.33%. For the AAT component, data for 54,948 animal blood samples were assembled from 218 geographic locations. The samples were tested with a variety of diagnostic methods. AAT was found in all surveyed departments, including the tsetse-free areas in the north. *Trypanosoma vivax* and *T. congolense* infections were the dominant ones, with a prevalence of 5.19 ± 18.97% and 6.11 ± 21.56%, respectively. *Trypanosoma brucei* infections were detected at a much lower rate (0.00 ± 0.10%).

**Conclusions:**

The atlas provides a synoptic view of the available information on tsetse and AAT distribution in Burkina Faso. Data are very scanty for most of the tsetse-free areas in the northern part of the country. Despite this limitation, this study generated a robust tool for targeting future surveillance and control activities. The development of the atlas also strengthened the collaboration between the different institutions involved in tsetse and AAT research and control in Burkina Faso, which will be crucial for future updates and the sustainability of the initiative.

**Graphical Abstract:**

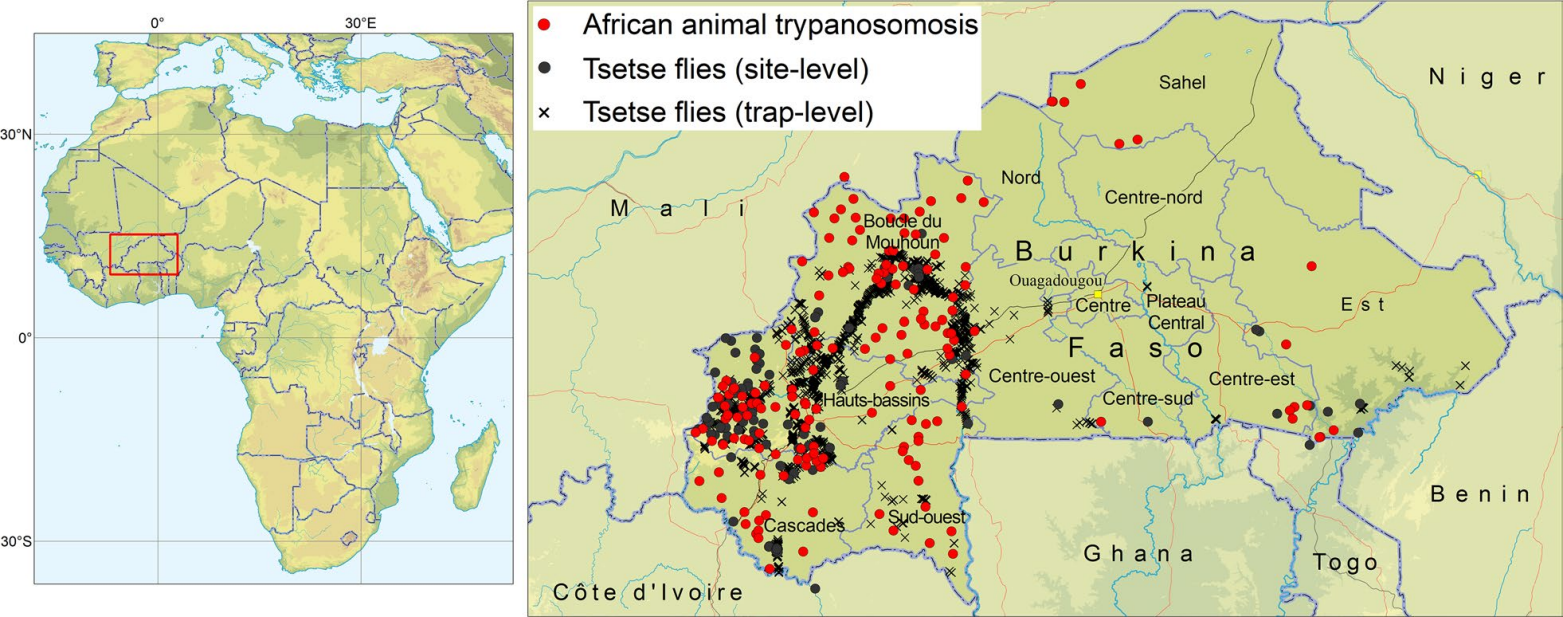

**Supplementary Information:**

The online version contains supplementary material available at 10.1186/s13071-021-05131-4.

## Background

Tsetse flies are the biological vectors of African trypanosomosis, a parasitic disease that affects both humans (sleeping sickness) [1] and animals (nagana) [2]. These diseases have historically hindered the development of the African continent [3], and they persist today as a major constraint to mixed crop-livestock agriculture, food security and human health [4]. The annual economic losses attributed to African animal trypanosomosis (AAT) are measured in billions of dollars [5, 6], while more than 50 million people are still considered at risk of contracting human African trypanosomosis (HAT) [7, 8].

In Burkina Faso, the expenditure on the trypanocides used for the preventive and curative treatment of livestock, mainly cattle, has been estimated at 3.9 million US$ per annum [9]. A total of 2.6 million doses of trypanocidal drugs are used every year to control the disease [9, 10], and 9 out of the 13 regions of the country are affected by tsetse flies. More than 60% of the country’s cattle population is raised in zones with high trypanosomosis risk [11, 12]. In particular, many zones where grazing potential for cattle is high and human activities are limited, also provide suitable habitats for tsetse flies [12].

In 2000, the Government of Burkina Faso, along with other African countries in the Pan-African Tsetse and Trypanosomosis Eradication Campaign (PATTEC), embarked on an initiative led by the African Union [13]. At the country level, a project in the PATTEC framework was implemented between 2006 and 2013 [14]. The project generated a vast amount of data, while other activities and projects implemented by other stakeholders also collected field data on the occurrence of AAT and its vectors in Burkina Faso. Unfortunately, the data generated by these activities and institutions have not been harmonized and centralized.

National atlases of tsetse and AAT are considered indispensable tools for efficient disease management in the context of the progressive control pathway (PCP) for AAT [15]. In 2013, the Food and Agriculture Organization of the United Nations (FAO) launched a multinational project to support six AAT-affected countries (i.e. Burkina Faso, Mali, Ghana, Kenya, Ethiopia and Uganda), and one of its goals was to promote the development of the national atlases. The Insectary of Bobo Dioulasso–Tsetse and Trypanosomosis Eradication Campaign (*Insectarium de Bobo Dioulasso-Campagne d'Eradication de la mouche Tsé-tsé et de la Trypanosomose*—IBD-CETT, formerly PATTEC Burkina Faso), implemented the FAO project in Burkina Faso as the specialized national structure in charge of tsetse and AAT control. IBD-CETT also took the lead in the development of the atlas.

## Methods

The methodology to develop the atlas of tsetse and AAT in Burkina Faso is broadly based on the FAO continental atlas [16–18], and a similar approach has already been used at the national level in Sudan [19], Mali [20], Kenya [21] and Zimbabwe [22]. The main difference between the continental and the national atlases is that the former is solely based on publicly available data from scientific journals, while the latter include all published or unpublished data collected in a given country.

The atlas in Burkina Faso covers a period of 30 years (1990–2019). Data were provided by all the institutions involved in tsetse and trypanosomosis research and control at the national level. In addition to IBD-CETT, these are the School of tsetse control (*Ecole de Lutte Anti-Tsé-tsé*—ELAT), the International Research and Development Centre on Livestock Farming in the Subhumid Zone (*Centre International de Recherche-Développement sur l'Elevage en zone Subhumide*—CIRDES), the French Agricultural Research Centre for International Development (*Centre de coopération Internationale en Recherche Agronomique pour le Développement*—CIRAD), the French Research Institute for Development (*Institut de Recherche pour le Développement*—IRD), the International Atomic Energy Agency (IAEA) and FAO.

### Input data

Input data were collected either in the framework of tsetse and trypanosomosis control operations or in the context of research activities. Control operations were mostly carried out by PATTEC Burkina Faso by using impregnated targets and traps and animal treatment. Data included both baseline cross-sectional surveys (i.e. pre-intervention) and longitudinal surveys (i.e. during or post-intervention), and provided a large amount of unpublished information. The raw data from research activities were obtained from the authors of published articles, using as a basis the systematic review of the literature carried out by FAO for the continental atlas. The full list of publications that contributed to the national atlas in Burkina Faso is provided in Additional file 1: Text S1. When the raw data underpinning publications could not be retrieved, information on AAT and tsetse occurrence was extracted directly from the papers, as done for the continental atlas.

### Tsetse data

In Burkina Faso, entomological surveys are mainly carried out with biconical traps [23], which may or may not be boosted with odour attractants [24]. However, other traps such as monoconical Vavoua trap [25] [26, 27], N’Zi traps, adhesive targets and electric targets were also occasionally used in experimental settings [28]. The trapping duration was usually 3 days for the surveys carried out in the framework of control activities, and 1 to 3 days for research activities. All traps were geo-referenced with GPS.

Entomological data were recorded at the level of individual traps. Data were initially captured in hard-copy recording sheets, which were subsequently converted into digital format (e.g. Microsoft Excel or Access). Field recording sheets include information such as the name of the surveyed location, its coordinates and administrative units (which in Burkina Faso are called regions, provinces and departments), the date of the survey, the time of trap deployment and removal (and the corresponding duration of trapping), and the number, species and sex of trapped tsetse flies. Complementary information (type of trap, use of odour attractants, etc.) is normally available in mission or project reports. For research activities linked to scientific publications, raw datasets at the trap level were normally obtained. When these were not available, information was mapped at the site level.

### African animal trypanosomosis data

As for tsetse, AAT data were mainly collected in the framework of control activities, thus including both baseline and monitoring/longitudinal investigations. Supplementary data were generated in the context of research.

AAT surveys in Burkina Faso target the main susceptible animal species such as cattle, sheep, goats and donkeys, but data on horses and camels are also occasionally collected. In most surveys, the sampling locations and the animals are selected randomly. However, a non-random sampling was sometimes performed, for example in studies of trypanocidal drug resistance [29, 30], or for the isolation of trypanosomes. Cross-sectional surveys provided the majority of datasets included in the atlas, but longitudinal investigations were also included [14].

Field data recording sheets for AAT include the data source and the institution in charge, the name, geographical coordinates and administrative units pertaining to the survey village or site, the date of the survey and the sample size (i.e. the number of animals tested). The sheets also include parasitological results such as the number of AAT-positive animals by trypanosome species, the haematocrit (packed cell volume—PCV) and the possible use of trypanocidal drugs (including the type of drug).

AAT data are recorded at the level of individual animal in the parasitological recording sheets, and information at the animal level was retained in the atlas. When the animal level data were not available, aggregated information at the herd/site level was recorded.

### Structure of the atlas

The atlas is composed of a structured data repository and two databases (tsetse distribution and AAT distribution). Data on tsetse infections are not yet included. Open-source PostgreSQL is used as the main database management system, together with its spatial extension PostGIS. Microsoft Access is used to facilitate a broader data utilization.

#### Data repository

The data repository includes digital copies of all the input files used to build the atlas, i.e. spreadsheets, databases, reports, thesis and scientific articles. At a first level, the repository is organized by type of data (i.e. tsetse or AAT), at a second level by geographical area (i.e. province), and last by individual data source or publication. File names for scientific publications are standardized as author name and year of publication (i.e. Surname_YEAR.pdf), and for unpublished works as title of activity and year.

#### Tsetse database

Entomological data recorded in the tsetse database are organized in six tables: 1) data sources; 2) geographical data; 3) entomological surveys (trap level); 4) tsetse catches (trap level); 5) entomological surveys (site level); and 6) tsetse catches (site level). As in all relational databases, records in each table are given a unique identifier, the ‘primary key’, which allows them to be linked to records in other tables through ‘foreign keys’.

The table on ‘data sources’ summarizes information on the input files stored in the repository. In particular, it records the following information: institution, author’s name, title, year of production/publication, presence of information on tsetse or AAT, availability of raw data and whether the source is published or not. The ‘geographical data’ table includes the location or village name, its geographical coordinates and the corresponding administrative units. The table on ‘entomological surveys’ (trap level) includes the code of the trap (as per survey recording sheet), the type of trap, the attractant used (if any), the geographical coordinates of the trapping site (latitude and longitude in decimal degrees on WGS84 datum), the trapping period and its duration. Information on the possible presence of tsetse control activities in the surveyed area is also recorded. The table on ‘tsetse catches’ (trap level) summarizes the results of the trapping and includes the tsetse species, sex and the number of flies caught.

The tables on ‘entomological surveys’ and ‘tsetse catches’ (site level) record data that were directly extracted from scientific publications when raw data at the trap level could not be obtained. These site-level tables of the national atlas for Burkina Faso have a similar structure to those of the continental atlas [17].

The detailed structure of the tsetse database, including the relationships between the tables, is provided in Additional file 2: Text S2.

#### African animal trypanosomosis database

The database for AAT contains seven tables: (1) data sources; (2) geographical data; (3) animal species; (4) diagnostic method; (5) AAT data (animal level); (6) AAT data (herd level); and (7) chemotherapy.

The tables ‘data sources’ and ‘geographical data’ are similar to those of the tsetse database. The table ‘animal species’ records the species and breed of tested animals, and the table ‘diagnostic method’ provides details on the diagnostic test used to detect AAT. The table ‘AAT data’ (animal level) includes the code of the animal (as per survey recording sheet), its age, sex, haematocrit/PCV, health status, treatment with trypanocidal drugs, date of last treatment, husbandry system, date of survey, presence or absence of trypanosomes, species of trypanosomes (including subspecies/groups), type of animal sampling (random or purposeful), type of survey (longitudinal or cross-sectional) and possible presence of vector control operations during the survey. The table ‘AAT data’ (herd level) is used to capture data extracted from scientific publications when the raw data at the animal level could not be obtained; as such, it follows the structure of the continental atlas [16]. The last table (chemotherapy) provides details on the possible treatment of animals with anti-trypanosomal drugs before the survey. It includes the date and cause of treatment, the drug used, the doses and the person who administered the treatment.

The detailed structure of the AAT database, including the relationships between the tables, is provided in Additional file 3: Text S3.

#### Development of the atlas

The first step in the development of the atlas was to collect input data from all partner institutions. Workshops and meetings were organized to facilitate the process. When input data could only be retrieved as hard copy, Microsoft Excel was used to enter data in digital format.

Merging data from a variety of sources into the atlas databases required a systematic process of verification and harmonization. Geographical coordinates were standardized as latitude and longitude (decimal degrees on WGS84 datum). When Universal Transverse Mercator (UTM) coordinates were reported in the input files, coordinate transformation was performed. Extensive harmonization was also needed for data on animal breeds, husbandry systems, names of geographical locations and the related administrative units. For tsetse data, the names of locations were often missing. For AAT data, a high proportion of epidemiological records lacked geographic coordinates; in these cases, the names of the survey sites were used to extract geographic coordinates from other sources (i.e. Geographical Institute of Burkina Faso, Google Earth and Google Maps). Also, input data sheets often lacked information on whether interventions against tsetse were taking place in the study area at the time of the survey, or whether trypanocidal drugs were being used. In these cases, reports and publications were often useful to fill the gaps. When raw data could not be retrieved, information was extracted directly from the papers; for example, the maps included in the publication were imported in the GIS and georeferenced to estimate the geographical coordinates of the study sites.

## Results

A total of 2261 data recording sheets were included in the atlas repository ( 723 for the tsetse component and 1538 for the AAT component). The review of the published and grey literature also enabled us to identify 105 eligible documents, including scientific papers, PhD and MSc theses, and activity reports.

**Fig. 1:**
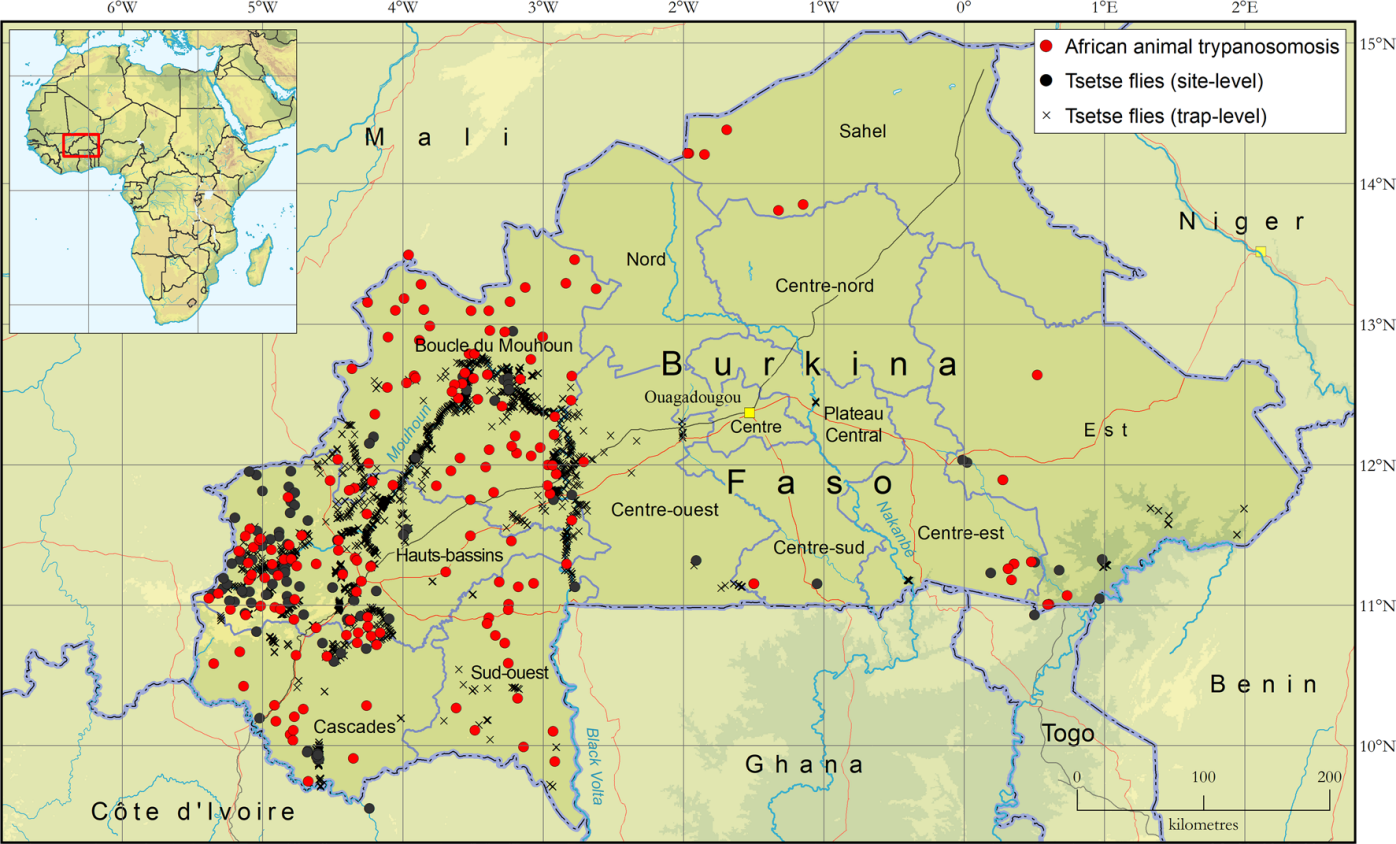
The occurrence of African animal trypanosomosis and tsetse flies (genus: *Glossina*) in Burkina Faso. Data collection period: 1990–2019

The results of the atlas are summarized in Fig. 1, which shows the locations where tsetse flies and AAT were detected in Burkina Faso during the period 1990–2019. Locations where surveys were conducted but tsetse and AAT were not detected are not shown in Fig. 1, although they are included in the database of the atlas.

### Tsetse fly distribution

Entomological data on tsetse fly distribution were assembled for a total of 19,960 tsetse trapping events in 10,022 distinct trapping locations, including 9768 locations at the trap level and 254 locations at the site level. ‘Site’ locations normally refer to groups of traps, and they were used to map data from publications when the underlying trap-level data could not be obtained. The overall trapping intensity was 48,345 trap days.

The bulk of the entomological data included in the atlas are from the western and south-western part of the country. In particular, intensive baseline and monitoring surveys were conducted between 2007 and 2013 in the region at the Loop of the Mouhoun River (*Boucle du Mouhoun*), in the framework of an attempted large-scale elimination campaign [14, 24]. Entomological surveys are limited in most of the other regions, and especially in the north-eastern parts of the country that are believed to be free of tsetse.

The device most commonly used to trap flies was the biconical trap [23], which was used in more than 95% of the surveys and often boosted with an odour attractant (77.31% of traps). Other trapping devices used were sticky targets [27], electric targets [28] and N’zi traps [31].

A total of 126,849 tsetse flies were caught in all the surveys included in the atlas, representing all three taxonomical groups (subgenera) historically present in Burkina Faso. The vast majority of catches belong to the palpalis group (91.91%), in particular *Glossina palpalis gambiensis* (35.56%) and *G. tachinoides* (56.35%). Flies of the morsitans group were represented by *G. morsitans submorsitans*, which accounted for 6.51% of the overall catches. *Glossina medicorum* of the fusca group accounted for 0.25% of the catches. Approximately 1.33% of flies collected were not identified to the species level*.*

**Fig. 2:**
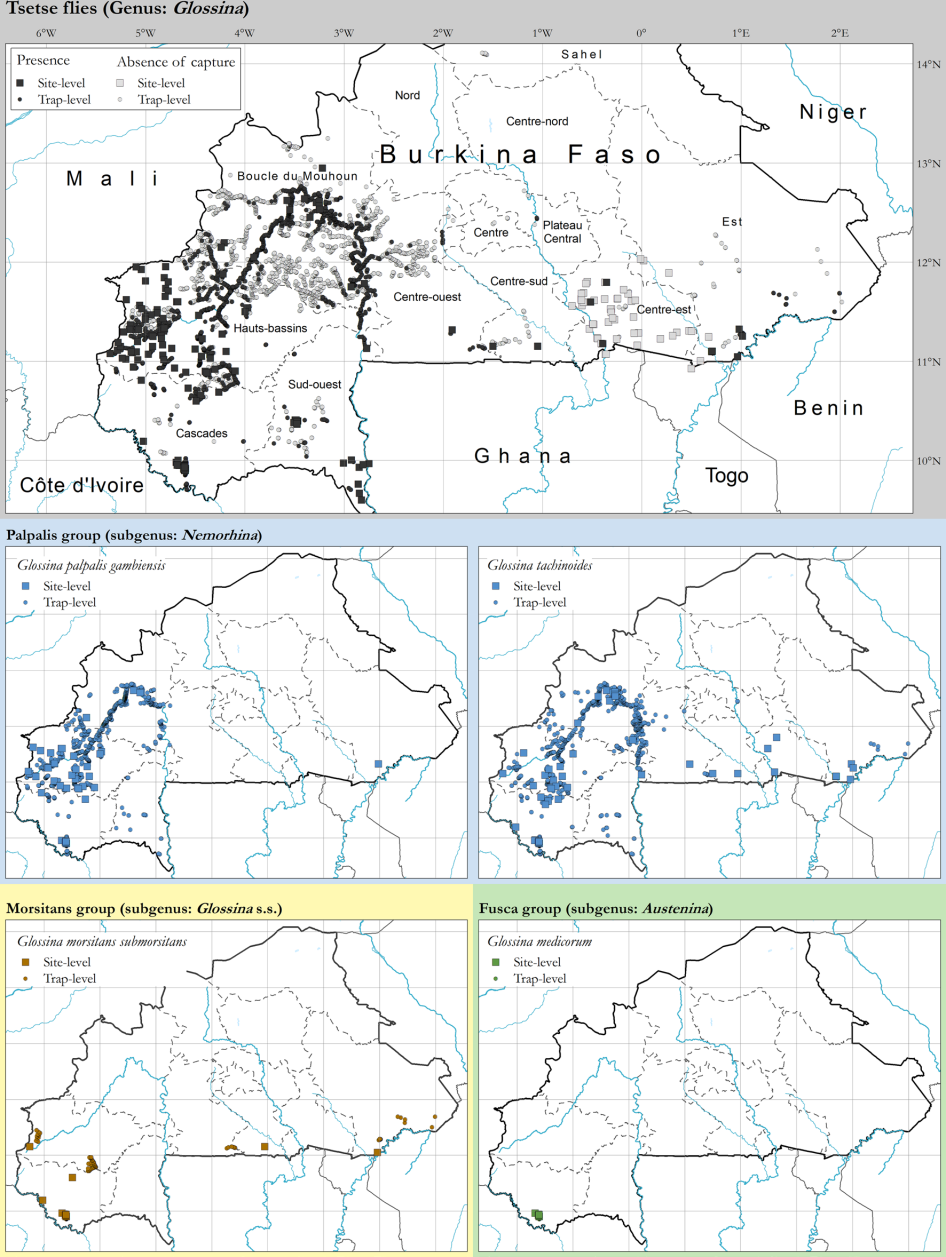
Presence and absence of capture of *G. palpalis gambiensis*, *G. tachinoides* and *G. morsitans submorsitans* and *G. medicorum* in Burkina Faso. Data collection period: 1990–2019

In terms of geographic distribution, *G. palpalis gambiensis* and *G. tachinoides* were found to be widespread in the western part of the country (Fig. 2), in particular along the major river systems (Mouhoun, Bourgouriba and Comoe) and their main tributaries. In the eastern part of the country, *G. palpalis gambiensis* was detected in only one location of Arly National Park, while *G. tachinoides* was found in several locations, and especially in protected areas (e.g. Nazinga Game Ranch and Arly National Park) [32]. The distribution of flies of the morsitans and fusca groups was more circumscribed. *Glossina morsitans submorsitans* was present in a few locations in the southern parts of the country, and strictly limited to protected areas, whereas *G. medicorum* was only found in one protected area in the south-east (Diefoula Forest and Logoniegue Forest).

### African animal trypanosomosis distribution

In the AAT component of the atlas, the results of AAT diagnosis for 54,948 animal’s blood samples were included, originating from 218 distinct geographic locations. Cattle account for 90.37% of animals tested (*n* = 49,654), followed by sheep (5.20%; *n* = 2859), goats (2.29%; *n* = 1258) and donkeys (2.10%; *n* = 1154). Very limited data were found on horses (*n* = 21) and camels (*n* = 2).

**Fig. 3:**
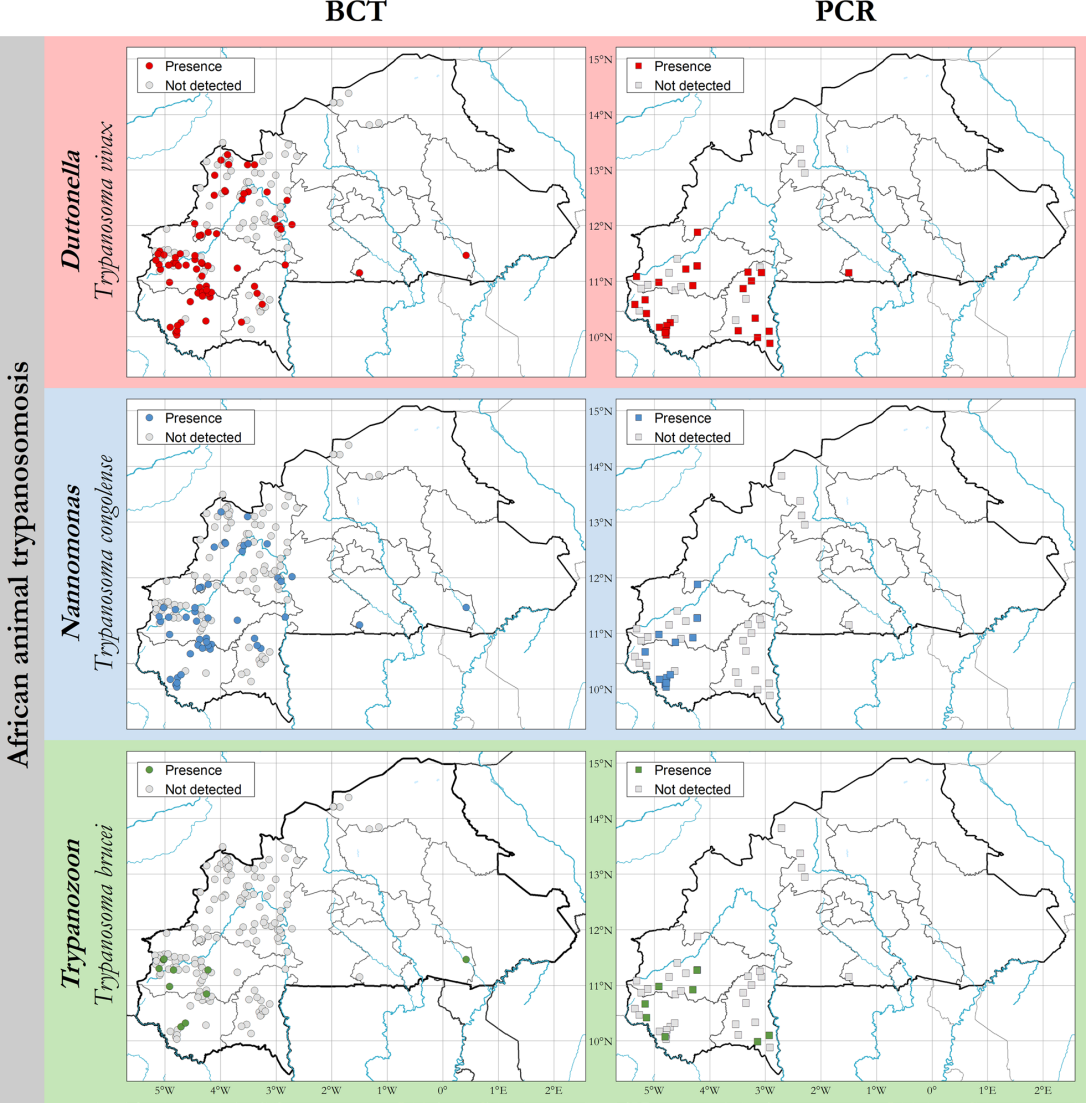
Presence and absence of African animal trypanosomosis in Burkina Faso. Data collection period: 1990–2019

AAT was confirmed to be present in all the tsetse-infested areas, in particular in the southern and south-western parts of the country. Hotspots of transmission appear to be located in the areas bordering Côte d’Ivoire and Mali, and in the Mouhoun River basin. To a lesser extent, transmission occurs in the eastern part of the country, especially in the areas bordering Togo and Benin near the Arli National Park (Fig. 3). Serological cases of the disease were also found in some of the tsetse-free areas in the northern part of the country [33], at a distance of over 200 km from the tsetse-infested belt [32]. However, very limited surveillance is carried out in these tsetse-free areas, and the true extent of the AAT problem in these regions cannot be ascertained with the available data.

**Fig. 4:**
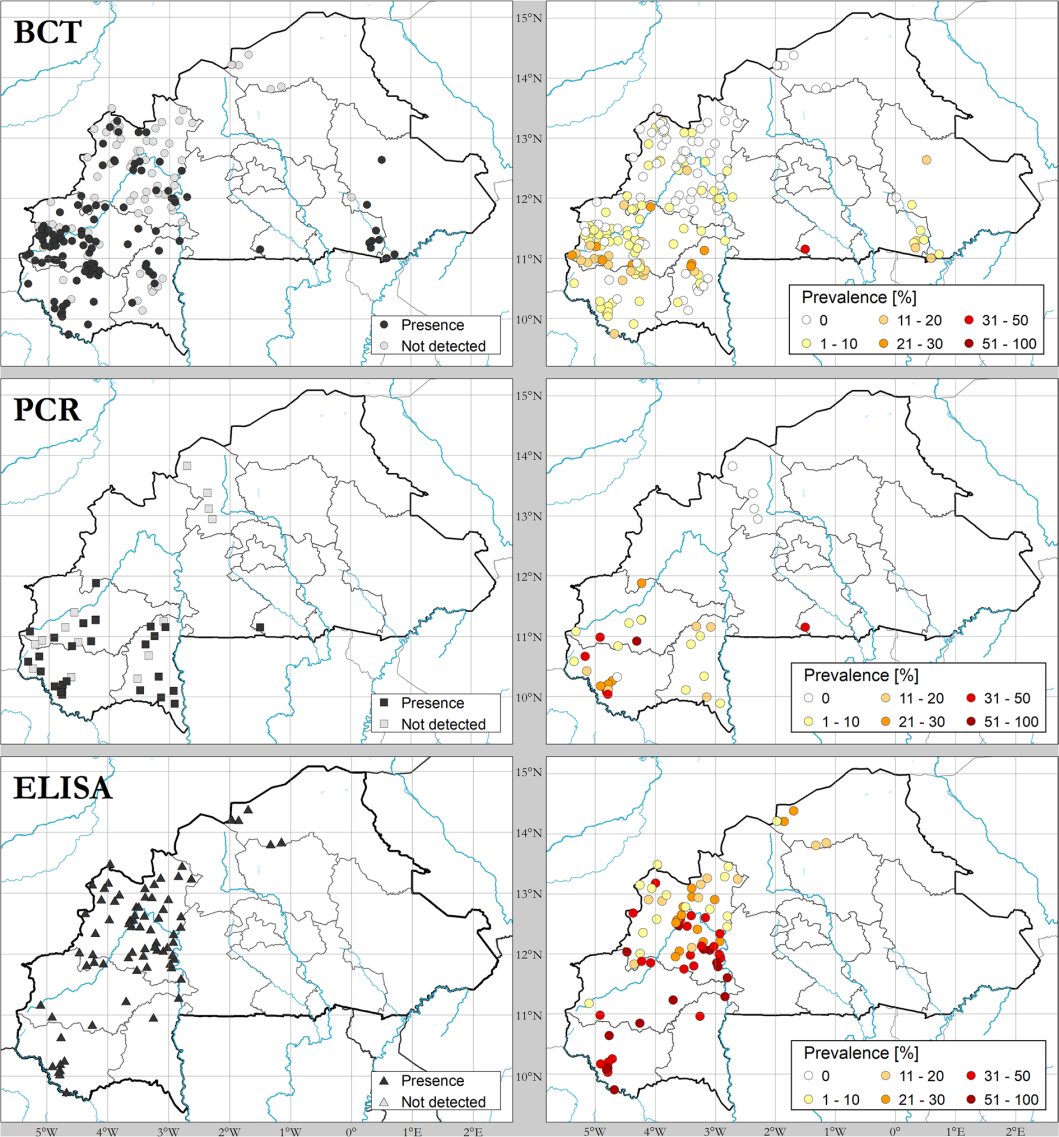
Presence and absence and prevalence of African animal trypanosomosis in Burkina Faso with different diagnostic methods. Data collection period: 1990–2019

The buffy coat technique (BCT) [34] was the diagnostic method most frequently used for the detection of trypanosomal infections (62.59% of all samples, *n* = 34,390), followed by serological techniques, more specifically enzyme-linked immunosorbent assay (ELISA) (28.63% of all samples, *n* = 15,733) [12, 35], and molecular techniques, in particular polymerase chain reaction (PCR) (8.78%, *n* = 4825) [36] (Fig. 4). Trypanosomosis prevalence was variable among hosts and diagnostic techniques. For cattle, an overall prevalence of 6.06% for BCT (1881/31,048), 45.83% for ELISA (6316/13,781) and 30.86% for PCR (1489/4825) was found (Table 1).

**Table 1:** Prevalence of AAT (*Trypanosoma* sp.) in different host species and by different diagnostic techniques in Burkina Faso (period: 1990 and 2019)

Diagnostic method	Animal species	Animals tested [*n*]	Number of infections [*n*]				Mean prevalence [%]				Standard deviation [%]			
			*Tc*	*Tv*	*Tb*	*T*	*Tc*	*Tv*	*Tb*	*T*	*Tc*	*Tv*	*Tb*	*T*
BCT	Donkey	673	5	7	0	12	0.74	1.84	0.00	2.58	4.82	10.70	0.00	11.79
	Cattle	31,048	712	642	7	1881	7.86	6.43	0.00	13.69	24.26	21.15	0.11	30.54
	Camels	1	0	0	0	0	0.00	0.00	0.00	0.00				
	Goats	801	2	6	0	8	0.10	0.40	0.00	0.50	0.78	2.82	0.00	3.28
	Horses	17	0	0	0	0	0.00	0.00	0.00	0.00	0.00	0.00	0.00	0.00
	Sheep	1850	2	18	0	20	0.05	0.97	0.00	1.03	0.84	4.92	0.00	5.05
Subtotal		34,390	721	673	7	1921	6.11	5.19	0.00	10.94	21.56	18.97	0.10	27.64
PCR	Cattle	2806	512	508	8	965	9.49	14.78	0.11	22.84	19.20	23.11	0.84	28.81
Elisa	Donkey	481	7	26	6	29	1.28	5.51	1.46	5.99	5.16	15.20	6.75	15.29
	Cattle	13,781	2444	4258	1069	6316	24.39	40.00	11.85	47.21	32.93	35.20	24.68	35.70
	Camels	1	1	1	1	1	100	100	100	100				
	Goats	457	5	25	16	43	0.82	4.84	2.13	7.48	3.51	13.95	6.09	15.76
	Horses	4	0	0	0	0	0.00	0.00	0.00	0.00	0.00	0.00	0.00	0.00
	Sheep	1009	98	162	48	203	6.01	9.48	2.27	12.40	15.22	23.68	7.56	25.81
Subtotal		15,733	2555	4472	1140	6592	18.78	31.17	9.26	37.03	30.28	34.94	21.97	36.55
Total	Donkey	1154	12	33	6	41	0.96	3.32	0.59	3.95	4.96	12.80	4.33	13.39
	Cattle	49,654	3747	5505	1105	9686	13.04	17.44	3.64	24.51	27.80	30.37	14.67	35.31
	Camels	2	1	1	1	1	50.00	50.00	50.00	50.00	70.71	70.71	70.71	70.71
	Goats	1258	7	31	16	51	0.39	2.18	0.85	3.29	2.32	9.33	3.98	10.82
	Horses	21	0	0	0	0	0.00	0.00	0.00	0.00	0.00	0.00	0.00	0.00
	Sheep	2859	100	180	48	223	2.32	4.21	0.86	5.36	9.83	15.65	4.79	17.29
General total		54,948	3867	5750	1176	10,002	10.47	14.32	3.02	20.12	25.24	28.14	13.23	33.05

Tc *T. congolense*, Tv *T. vivax*; Tb: *T. brucei*, *T* number of animals infected, *[n]* number, *[%]* prevalence

Three species of trypanosomes pathogens for livestock, *Trypanosoma vivax*, *T. congolense* and *T. brucei*, were found. Parasitological techniques showed an overall prevalence of 5.58% (1921 infections/34,390) and 30.68% (1489 infections/4825) for molecular techniques, while serological technique showed an overall prevalence of 41.90% (6592 infections/15,733). *Trypanosoma vivax* and *T. congolense* were the most common species responsible for AAT. The parasitological prevalence by BCT for *T. vivax* was 5.19 ± 18.97% (673 infections) and 6.11 ± 21.56% (721 infections) for *T. congolense*. *Trypanosoma brucei* was detected at smaller rates (0.00 ± 0.10; 7 infections). With PCR, the prevalence was 14.78 ± 23.11% and 9.49 ± 19.20% for *T. vivax* and *T. congolense*, respectively. The prevalence of *T. brucei* was 0.11 ± 0.84% (Table 1).

### Database completeness

A high level of completeness was achieved in the atlas database. Regarding the tsetse component, the required information items could be found for the vast majority of the entomological records, in particular trap coordinates (83.57%), administrative units (100%) [i.e. region, province, department], location names (89.69%), date of trapping (99.52%), duration of trapping (97.25%), type of trap (99.25%), tsetse species (98.65%), tsetse apparent densities (97.25%) and control interventions against tsetse (76.34%). The majority of trap coordinates were in UTM (75.72%), so they had to be converted into geographical coordinates (Decimal Degrees). Regarding the AAT component, a similarly high level of completeness was achieved for geographical coordinates (87.12%), administrative units (90.72), location names (90.49%), survey period (97.40%), sample size (100%), number and species of infected animals (84.89%), sampling strategy (i.e. random or purposeful) (95.93%), chemotherapy (67.77%) and possible interventions against tsetse (84.37%).

## Discussion

Our review of the published and unpublished data provides a synoptic picture of the distribution of tsetse fly and AAT in Burkina Faso for the period 1990–2019. The atlas updates the latest national tsetse distribution map produced in 1977 [37], whose northern limit had been updated in 2010 [32]. Distribution maps of the four tsetse species present in Burkina Faso are also presented. As far as AAT is concerned, the distribution maps presented in this paper, based on the testing of over 50,000 animals, are the first of their kind in Burkina Faso.

However, as observed in similar national mapping exercises [19–22], a number of gaps not attributable to the data compilation and processing still affect our knowledge of the distribution of tsetse fly and AAT in Burkina Faso. The gaps are related to the inherent limitations of the existing datasets, and in particular to the geographical coverage, range of animal species tested and diagnostic methods.

With regard to the geographical coverage, the main gap in the atlas is the severe lack of information from the north, north-east, north-west, centre and extreme east of the country. This situation is explained by the fact that institutions involved in tsetse and AAT research and control in Burkina Faso focus their investigations and efforts on the regions of highest tsetse infestation [14, 38]). On the other hand, the northern and eastern regions are plagued by insecurity, which severely limits field data collection activities in these areas. As already observed in Mali [20], this lack of information for a large part of the national territory hides the true extent of the AAT problem in the country.

As regards the animal species tested for AAT, more than 90% of the atlas data are on cattle. However, in Burkina Faso small ruminants (i.e. sheep and goats) far exceed cattle in terms of population numbers. Furthermore, the diagnostic technique used to test blood samples was the BCT for more than 60% of the samples. While parasitological techniques based on blood concentration may be appropriate for treatment decisions [39], they are known to have low sensitivity and to underestimate the true prevalence of the disease [35].

Despite the limitations, the atlas provides a fairly accurate assessment of the geographic distribution of three tsetse species of veterinary importance in Burkina Faso: *G. palpalis gambiensis, G. tachinoides* and *G. morsitans submorsitans*. *Glossina medicorum*, because of its limited distribution, has a very low impact on AAT transmission in Burkina Faso. A fifth species, *G. longipalpis*, had been reported in Burkina Faso, in particular in the south-west region in the zone of Batié [37]. However, the last observation was in 1961 [37], and recent data from this region are insufficient to rule out its possible persistence.

Thanks to their capacity to adapt to land cover changes and vegetation degradation, tsetse species of the riverine group (i.e. palpalis group) are confirmed to be the most widespread in Burkina Faso [37]. In particular, the geographical range of *G. tachinoides* extends from the east to the west of the country, and the species is now also found in some previously free areas (e.g. western part of the Orodara department) [37].

As regards savannah species, their distribution is fragmented and confined to a few protected areas. This pattern has been observed in other countries in western Africa [18], as a result of habitat degradation, land cover changes and the depletion of the wild fauna, their preferred hosts [40]. *Glossina morsitans submorsitans* is found along the southern border between Burkina Faso and Ghana and Benin, as well as in the pastoral zones in the western part of the country (Samorogouan and Sideradougou). *Glossina medicorum* was reported from one province in the south-western part of the country (Comoé), while it had also been previously reported along of the borders with Ghana, Togo and Benin [37].

Regarding animal trypanosomosis, the three major trypanosome species infective for livestock (*T. vivax*, *T. congolense* and *T. brucei*) are widespread in the country. Serologically positive animals, not confirmed parasitologically, have also been reported from the north of the country (Djibo department) [33]. The absence of parasitological confirmations in these areas is ascribed to the combined effect of the absence of biological vectors and the trypanocidal drug treatments administered by livestock keepers before, during and after transhumance in the AAT enzootic area [33]. In this context, it is unclear whether any of the serologically positive animals identified in the northern area have been infected locally.

In Burkina Faso, *T. vivax* is the most widespread trypanosome species, followed by *T. congolense*. This can be ascribed to different factors, including the vectorial competence of different tsetse flies [41–44], and mechanical transmission by haematophagous flies such as *Stomoxys* and *Tabanids* [45]. Indeed, *T. vivax* is typically effectively transmitted by tsetse flies of the palpalis group, which are prevailing in Burkina Faso. Conversely, *T. congolense* and *T. brucei* are more frequently transmitted by tsetse of the morsitans group, which in Burkina Faso are now restricted to a few protected areas. Mechanical transmission also plays a role in the relative abundance of trypanosome species because, even though mechanical transmission of *T. congolense* is possible [45], this mode of transmission is normally much more important for *T. vivax*. Indeed, in some contexts, mechanical transmission is known to act as an amplifier of AAT transmission [46–48].

As to *T. brucei*, it was found mainly confined to protected areas in the west, south-west and centre-east of the country. These areas are of economic interest to the country, as they include pastoral areas, a park and a reserve.

In the development of our atlas, no data on *T. evansi* infections were retrieved. *Trypanosoma evansi* is the causative agent of ‘surra’, and its occurrence in Burkina Faso is mentioned in a narrative review by Desquesnes et al. [49]. However, a recent systematic literature review did not identify any reference to this effect [50]. Indeed, in Africa *T. evansi* is mainly reported from dromedary camels, a host species for which very limited data are available in Burkina Faso. Similar considerations apply to the absence of data on pigs and the absence of reports on *T. simiae* infections.

The pooled prevalence of AAT in different hosts varies depending on animal species and the detection methods used. The prevalence is higher in cattle than in the other livestock species. This can be ascribed to the predominantly extensive cattle breeding system, which relies on natural pastures. Higher AAT prevalence was reported with serological techniques, which is mainly due to the persistence of the antibodies over several months after curative treatment or the possibility of low undetectable parasitaemia in parasitological techniques [51–54].

## Conclusion

The main achievement of the present study was to compile and georeference most of the available data on tsetse and AAT occurrence in Burkina Faso for a 30-year period. It also allowed us to generate updated maps of tsetse and AAT distribution at the national level. Our data show that animal trypanosomosis is widespread in Burkina Faso, even though tsetse flies are mainly confined to the west, south-west and east of the country. The disease therefore poses a constraint to the development of the livestock and agricultural sectors at the national level. The prevalence of AAT is highest in the west, which is considered the breadbasket of the country. The newly developed national atlas represents a valuable tool to support the progressive control of AAT at the national level, in particular to target and monitor surveillance and control activities. The development of the atlas also helped to develop capacities for data management of all the involved partners. It promoted synergies among national institutions for data collection and sharing, and the establishment of mechanisms for regular updating and upgrading. With a view to filling the present geographical gaps, AAT data collection could include the east, north-east and the centre where data are not available. Entomological surveys could also include the Batié area to update the status of *G. longipalpis*. Additional work may be carried out to include tsetse fly infections in the atlas.

## Supplementary Information

**Additional file 1: Text S1**. List of scientific publications that contributed to developing the national atlas of tsetse and African animal trypanosomosis in Burkina Faso.

**Additional file 2: Text S2**. Structure of the tsetse database

**Additional file 3: Text S3**. Structure of the African animal trypanosomosis database

## Data Availability

Relevant data are within the paper and its additional files. The bulk of the data of the atlas of tsetse and AAT in Burkina Faso is the property of the Government of Burkina Faso, *Ministère des Ressources Animales et Halieutiques*, *Insectarium de Bobo-Dioulasso–Campagne d’Eradication de la Lutte contre les Mouches tsé-tsé et de la Trypanosomose*, (IBD-CETT). Data can be requested from: Director General, PO Box: 1087, Bobo-Dioulasso 01, Burkina Faso, Phone: +226 20 970955/971521, website: www.pattec.bf.

## Abbreviations

AAT: African animal trypanosomosis
AfDB: African Development Bank
BCT: Buffy coat technique
PCR: Polymerase chain reaction
IBD-CETT: Insectarium de Bobo-Dioulasso–Campagne d’Eradication de la Lutte contre les Mouches tsé-tsé et de la Trypanosomose (Coordination Unit for Tsetse and Animal Trypanosomosis Control)
FAO: Food and Agriculture Organization of the United Nations
IRD: Institut de recherche pour le développement
CIRAD: Centre de coopération internationale en recherche agronomique pour le développement
CIRDES: Centre International de Recherche-Développement sur l'Elevage en zone Subhumide
ELAT: Ecole de Lutte anti-tsetse
GPS: Global Positioning System
HAT: Human African trypanosomosis
IAEA: International Atomic Energy Agency
PATTEC: Pan-African Tsetse and Trypanosomosis Eradication Campaign
PCP: Progressive control pathway
PCV: Packed cell volume
UTM: Universal Transverse Mercator
WGS84: World Geodetic System 1984
