# Supplementary material for: An atlas to support the progressive control of tsetse-transmitted animal trypanosomosis in Burkina Faso
Source: Parasit Vectors. 2022 Mar 4;15:72. doi: 10.1186/s13071-021-05131-4 (PMC8895521; doi:10.1186/s13071-021-05131-4)
Supplement: Supplementary file 1 — Additional file 1: Text S1. List of scientific publications that contributed to developing the national atlas of tsetse and African animal trypanosomosis in Burkina Faso. [file 13071_2021_5131_MOESM1_ESM.docx]

# Additional file 1: Text S1

# List of scientific publications that contributed to developing the national atlas of tsetse and African animal trypanosomosis in Burkina Faso

1. Sophie Ravel, Jean-Baptiste Rayaisse, Fabrice Courtin, Philippe Solano, Thierry de Meeus. Genetic signature of a recent southern range shift in Glossina tachinoides in East Burkina Faso*. Infect, Genet Evol* 18 (2013) 309–314
2. Pagabeleguem S, Sangaré M, Bengaly Z, Akoudjin M, Belem AM, Bouyer J. Climate, Cattle Rearing Systems and African Animal Trypanosomosis Risk in Burkina Faso. *PLoS ONE* (2012) 7(11): e49762.
3. L. Percoma, A. Sow, S. Pagabeleguem, Ahmadou H. Dicko, O. Serdebéogo, M. Ouédraogo et al. Impact of an integrated control campaign on tsetse populations in Burkina Faso. *Parasites & Vectors* (2018) 11:270
4. L. Percoma, Z. Koudougou, O. Serdebeogo, I. Tamboura, M. Ouedraogo, J. Bouyer, et al. Enquetes entomologiques preparatoires à une lutte à grande échelle contre les glossines, assistées par un système d’information géographique : cas de la Pattec au Burkina Faso. *Rev. Élev méd. Vét. Pays trop*., 2015, 68 (4) : 157-165
5. Rayaisse JB, Tirados I, Kaba D, Dewhirst SY, Logan JG, Diarrassouba A, and al. Prospects for the Development of Odour Baits to Control the Tsetse Flies (2010). *PLoS Negl Trop Dis* 4(3): e632.
6. Rayaisse JB, Esterhuizen J, Tirados I, Kaba D, Salou E, Diarrassouba A and al. Towards and Optimal Design of Target for Tsetse Control: Comparisons of Novel Targets for the Control of Palpalis Group Tsetse in West Africa. *PLoS Negl Trop Dis* 5(9): e1332.
7. Rayaisse JB, Salou E, Kiema S, Akoudjin M, Kaba D, Kagbadouno M and al. Tsetse diversity and abundance in Southern Burkina Faso in relation with the vegetation. *Parasitol Res. 2015 Sep*;114(9):3357-63.
8. Bouyer J, Guerrini L., G. Cesar J., De La Rocque, S. & Cuisance, D. A phyto-sociological analysis of the distribution of riverine tsetse flies in Burkina Faso. *Med Vet Entomol*. 2005 Dec;19(4):372-8.
9. De Meeus T, Ravel S, Rayaisse JB, Courtin F, Solano P. Understanding local population genetics of tsetse: The case of an isolated population of Glossina palpalis gambiensis in Burkina Faso. *Infect Genet Evol*. 2012 Aug;12(6):1229-34.
10. Courtin F, Rayaisse JB, Tamboura I, Serdebeogo O, Koudougou Z, Solano P, Sidibe I. Updating the Northern Tsetse Limit in Burkina Faso (1949–2009): Impact of Global Change. *Int J Environ Res Public Health*. 2010 Apr;7(4):1708-19.
11. Bouyer J, Stachurski F, Gouro AS, Lancelot R. Control of bovine trypanosomosis by restricted application of insecticides to cattle using footbaths*. Vet Parasitol*. 2009 May 12;161(3-4):187-93.
12. Bouyer J, Ravel S., Guerrini L., Dujardin J. P., Sidibe I., Vreysen M. Jand al. Population structure of *Glossina palpalis gambiensis* (Diptera: Glossinidae) between river basins in Burkina Faso: Consequences for area-wide integrated pest management. *Infect Genet Evol*. 2010 Mar;10(2):321-8.
13. Koné N, De Meeus T., Bouyer J., Ravel S., Guerrini L., N'Goran E. K. & Vial, L., Population structuring of the tsetse *Glossina tachinoides* resulting from landscape fragmentation in the Mouhoun River basin, Burkina Faso. *Med Vet Entomol.* 2010 Jun;24(2):162-8.
14. Sedda L, Guerrini L, Bouyer J, Koné N, Rogers DJ. Spatio-temporal modelling of *Glossina palpalis gambiensis* and *Glossina tachinoides* apparent densities in fragmented ecosystems of Burkina Faso. *Ecography* 33: 772-783, 2010.
15. Bouyer J, Koné N, Bengaly Z. Dynamics of tsetse natural infection rates in the Mouhoun river, BurkinaFaso,in relation with environmental factor. *Front Cell Infect Microbiol.* 2013 Aug 29;3:47.
16. Koné N, N'goran EK, Sidibe I, Kombassere AW, Bouyer J. Spatio-temporal distribution of tsetse and other biting ﬂies in the Mouhoun River basin, Burkina Faso. *Med Vet Entomol.* 2011 Jun;25(2):156-68.
17. Koné N, Bouyer J, Ravel S, Vreysen MJ, Domagni KT, Causse S, and al. Contrasting Population Structures of Two Vectors of African Trypanosomoses in Burkina Faso: Consequences for Control. *PLoS Negl Trop Dis.* 2011 Jun;5(6):e1217.
18. Guerrini L, Bouyer J, Mapping African animal trypanomosis risk: the landscape approach. *Vet Ital.* Jul-Sep 2007;43(3):643-54.
19. Guerrini L, Bord, J. P., Ducheyne, E. et Bouyer, J. Fragmentation Analysis for Prediction of Suitable Habitat for Vectors: Example of Riverine Tsetse Flies in Burkina Faso. *J Med Entomol.* 2008 Nov;45(6):1180-6.
20. Sow A, Ganaba R, Percoma L, Sidibe I, Bengaly Z, Adam Y, and al. Baseline survey of animal trypanosomosis in the region of the Boucle du Mouhoun, Burkina Faso. *Res Vet Sci.* 2013 Jun;94(3):573-8.
21. Dayo GK, Bengaly Z, Messad S, Bucheton B, Sidibe I, Cene B. and al. Prevalence and incidence of bovine trypanosomosis in an agro-pastoral area of southwestern Burkina Faso. *Res Vet Sci.* 2010 Jun;88(3):470-7.
22. Sidibe M, Boly H, Lakouetené T, Leroy P, Bosma RH. Characteristics of peri-urban Dairy Herds of Bobo-Dioulasso, Burkina Faso. *Trop Anim Health Prod.* 2004 Jan;36(1):95-100.
23. Sow A, Sidibe I., Desquesnes M., Bengaly, Z. & Pangui, L.J. The application of PCR-ELISA to the detection of *Trypanosoma congolense* type savannah (TCS) in bovine blood samples. *Trop Biomed.* 2006 Jun ;23(1) :123-9.
24. Silbermayr K, Li F, Soudré A, Müller S, Sölkner J. A Novel qPCR Assay for the Detection of African Animal Trypanosomosis in Trypanotolerant and Trypanosusceptible Cattle Breeds. *PLoS Negl Trop Dis.* 2013 Aug 15 ;7(8): e2345.
25. Desquesnes M, Z. Bengaly, M.L. Dia. Evaluation de la persistance des anticorps détectés par Elisa-indiect *Trypanosoma vivax* après traitement trypanocide chez des bovins naturellement infectés. *Rev. Elev. Méd. Vét. Pays trop.* ***2003***, 56(3-4), p. 141-144.
26. Yvonne Gall, Tanja Woitag, Burkhard Bauer, Issa Sidibe, John McDermottc, Dieter Mehlitz, Peter-Henning Clausen,. Trypanocidal failure suggested by PCR results in Cattle field samples. *Acta Trop.* 2004 Sep;92(1):7-16.
27. Sow A, Sidibé I, Bengaly Z, Marcotty T, Séré M, Diallo A, and al. Field detection of resistance to isometamidium chloride and diminazene aceturate in *Trypanosoma vivax* from the region of the Boucle du Mouhoun in Burkina Faso. *Vet Parasitol.* 2012 Jun 8; 187(1-2):105-11.
28. **Reifenberg Jean-Marc, Solano Philippe, Bauer Burkhard, Kaboré Idrissa, Cuny Gérard, Duvallet Gérard, Cuisance Dominique**. Apport de la technique PCR pour une meilleure compréhension de l'épizootiologie des trypanosomoses bovines : exemple de la zone d'aménagement pastoral de Yalé au Burkina Faso. Revue d'Elevage et de Médecine Vétérinaire des Pays Tropicaux (1997), 50 (1): 14-22.
29. Lefrançois Thierry, Solano Philippe, De La Rocque Stéphane, Bengaly Zakaria, Reifenberg Jean-Marc, Kaboré Idrissa, Cuisance Dominique. [New epidemiological features on animal trypanosomiasis by molecular analysis in the pastoral zone of Sideradougou, Burkina Faso.](http://agritrop.cirad.fr/390661/) Molecular Ecology (7) : 897-904 ; 1998. <https://doi.org/10.1046/j.1365-294x.1998.00420.x>
30. **Pierre** Fauret**, Charles** Dayo**, Jean-Baptiste** Rayaisse**, Sié Herman** Pooda**, Fabien** Dofini**, Philippe** Salano**, Bernard** Calas **et Fabrice** Courtin, « Dynamiques de peuplements, modifications environnementales et variation du risque trypanosomien dans le sud-ouest du Burkina Faso de 2005 à 2014 », Dynamiques environnementales, 36 | 2015, 146-165.
31. Sow Adama, Ouédraogo Sayouba, Sidibé Issa, Kalandi Miguiri, Zabré Marcelin, Sawadogo Germain. Enquête de base parasitologique de la trypanosomose animale dans trois zones agropastorales du Burkina Faso. *Bull. Anim. Hlth. Prod. Afr., (2014), 62, 241-250*
32. Bengaly Zakaria, Ganaba Rasmané, Sidibé Issa, Desquesnes Marc. Trypanosomose animale chez les bovins dans la zone Sud-soudanienne du Burkina Faso. Résultats d’une enquête sérologique Revue d'Elevage et de Médecine Vétérinaire des Pays Tropicaux, (2001)*, 54(3-4): p221-224. DOI:10.19182/remvt.9777*.
33. Bengaly Zakaria, Ganaba Rasmané, Sidibé Issa et G. Duvallet. Infections trypanosomiennes chez des bovins dans la zone Sud-soudanienne du Burkina Faso. *Revue Élev. Méd. vét. Pays trop., 1998, 51 (3) : p225-229*.
34. Amsler Sandrine, Filledier J. Comparaison de différents systèmes de collecte avec deux types de pièges pour la capture des glossines et des Tabanidés. *Revue d'Elevage et de Médecine Vétérinaire des Pays Tropicaux*, 1994, **47** (4) : 387-396.
35. McDermott, John J., Woitag, T., Sidibe, I., Bauer, B., Diarra, B., Ouédraogo, D., Kamuanga, M., Peregrine, A.S., Eisler, M.C., Zessin, K.H. Field studies of drug-resistant cattle trypanosomes in Kenedougou Province, Burkina Faso, Acta Tropica; 86(1): (2003) 93-103
36. M. Desquesnes, J.F. Michel S. de La Rocque, P. Solano, L. Millogo, Z. Bengaly, I. Sidibe. Enquête parasitologiqueet sérologique (Elisa-indirect) sur les trypanosomoses des bovins dans la zone de Sidéradougou, Burkina Faso. *Revue Élev. Méd. vét. Pays trop. 1998, 51 (3) : 225-229.*
37. [S. Amsler](https://www.semanticscholar.org/author/S.-Amsler/89036332), [J. Filledier](https://www.semanticscholar.org/author/J.-Filledier/6992220), [R. Millogo](https://www.semanticscholar.org/author/R.-Millogo/90722891). Efficacité comparée de différents pièges pour la capture de *Glossina tachinoides* (Diptera : Glossinidae) au Burkina Faso. *Revue Élev. Méd. vét. Pays trop., 1994, 47 (2) : 207-214*
38. Sandrine Amsler-Delafosse, Idrissa Kabore, Burkhard Bauer. Lutte contre les vecteurs de la trypanosomose animale africaine au Burkina Faso. Cahiers Agriculture, 1995 ; 4 : 440-3.
39. [Antonios A. Augustinos](https://bmcmicrobiol.biomedcentral.com/articles/10.1186/s12866-018-1295-4#auth-Antonios_A_-Augustinos), [Irene K. Meki](https://bmcmicrobiol.biomedcentral.com/articles/10.1186/s12866-018-1295-4#auth-Irene_K_-Meki), [Guler Demirbas-Uzel](https://bmcmicrobiol.biomedcentral.com/articles/10.1186/s12866-018-1295-4#auth-Guler-Demirbas_Uzel), [Gisele M. S. Ouédraogo](https://bmcmicrobiol.biomedcentral.com/articles/10.1186/s12866-018-1295-4#auth-Gisele_M__S_-Ou_draogo), [Aggeliki Saridaki](https://bmcmicrobiol.biomedcentral.com/articles/10.1186/s12866-018-1295-4#auth-Aggeliki-Saridaki), [George Tsiamis](https://bmcmicrobiol.biomedcentral.com/articles/10.1186/s12866-018-1295-4#auth-George-Tsiamis), [Andrew G. Parker](https://bmcmicrobiol.biomedcentral.com/articles/10.1186/s12866-018-1295-4#auth-Andrew_G_-Parker), [Adly M. M. Abd-Alla](https://bmcmicrobiol.biomedcentral.com/articles/10.1186/s12866-018-1295-4#auth-Adly_M__M_-Abd_Alla) & [Kostas Bourtzis](https://bmcmicrobiol.biomedcentral.com/articles/10.1186/s12866-018-1295-4#auth-Kostas-Bourtzis). Nuclear and *Wolbachia*-based multimarker approach for the rapid and accurate identification of tsetse species. BMC Microbiology 2018, 18(Suppl 1):147
40. Bouyer Jérémy, Guerrini Laure, Desquesnes Marc, De La Rocque Stéphane, Cuisance Dominique. Mapping african animal trypanosomosis risk from the sky. *Veterinary Research*, 2006, 37 : 633-645.
41. Jérémy Bouyer. Does isometamidium chloride treatment protect tsetse flies from trypanosome infections during SIT campaigns? *Medical and Veterinary Entomology* (2008) **22**, 140–143*.*
42. Bouyer, Jérémy et al "Mapping landscape friction to locate isolated tsetse populations that are candidates for elimination." *Proceedings of the National Academy of Sciences 112.47 (2015): 14575-14580. Web. 18 May. 2021*.
43. Dama E., Cornélie Sylvie, Somda M. B., Camara M., Kambire R., Courtin Fabrice, Jamonneau Vincent, Demettre E., Seveno M., Bengaly Z., Solano Philippe, Poinsignon Anne, Remoué Franck, Belem A. M. G., Bucheton Bruno. Identification of *Glossina palpalis gambiensis* specific salivary antigens: towards the development of a serologic biomarker of human exposure to tsetse flies in West Africa. *Microbes and Infection*, (2013), 15 (5), p. 416-427. ISSN 1286-4579.
44. **De La Rocque Stéphane, Augusseau Xavier, Guillobez Serge, Michel Vincent, De Wispelaere Gérard, Bauer Burkhard, Cuisance Dominique**. The changing distribution of two riverine tsetse flies over 15 years in an increasingly cultivated area of Burkina Faso. Bulletin of Entomological Research, 2001, **91** (3) : 157-166.
45. S. de La Rocque, J.F. Michel, J. Bouyer, G. De Wispelaere, D. Cuisance. Geographical Information Systems in parasitology: a review of potential applications using the example of animal trypanosomosis in West Africa. *Parassitologia*. 2005 Mar;47(1):97-104.
46. De Meeûs T, Ravel S, Rayaisse JB, Kaba D, Courtin F, Bouyer J, Dayo GK, Camara M, Solano P. Genetic correlations within and between isolated tsetse populations: what can we learn? Acta Trop. 2014 Oct;138 Suppl:S6-11. doi: 10.1016/j.actatropica.2014.03.004. Epub 2014 Mar 19. PMID: 24657846.
47. Dicko AH, Percoma L, Sow A, Adam Y, Mahama C, Sidibé I, et al. A Spatio-temporal Model of African Animal Trypanosomosis Risk. PLoS Negl Trop Dis, (2015) 9(7): e0003921.
48. V. Doudoumis, F. Blow, A. Saridaki, A. Augustinos, N. A. Dyer, I. Goodhead , P. Solano, J.-B. Rayaisse, P. Takac, S. Mekonnen, A. G. Parker, A. M. M. Abd-Alla, A. Darby, K. Bourtzis& G. siamis. Challenging the Wigglesworthia, Sodalis, Wolbachia symbiosis dogma in tsetse flies: Spiroplasma is present in both laboratory and natural populations. Scientific **Reports** | 7: *4699* | DOI:10.1038/s41598-017-04740-3
49. Esterhuizen J, Rayaisse JB, Tirados I, Mpiana S, Solano P, Vale GA, Lehane MJ, Torr SJ. Improving the cost-effectiveness of visual devices for the control of riverine tsetse flies, the major vectors of human African trypanosomiasis. PLoS Negl Trop Dis. 2011 Aug;5(8):e1257. doi: 10.1371/journal.pntd.0001257. Epub 2011 Aug 2. PMID: 21829743; PMCID: PMC3149014.
50. Hoppenheit A, Murugaiyan J, Bauer B, Clausen PH, Roesler U. Analysis of *Glossina palpalis gambiensis* and *Glossina tachinoides* from two distant locations in Burkina Faso using MALDI TOF MS. Parasitol Res. 2014 Feb;113(2):723-6. doi: 10.1007/s00436-013-3701-z. Epub 2013 Nov 29. PMID: 24292542.
51. Lefrançois T, Solano P, Bauer B, Kabore I, Touré SM, Cuny G, Duvallet G. Polymerase chain reaction characterization of trypanosomes in *Glossina morsitans submorsitans* and *G. tachinoides* collected on the game ranch of Nazinga, Burkina Faso. Acta Trop. 1999 Jan 15;72(1):65-77. doi: 10.1016/s0001-706x(98)00080-1. PMID: 9924962.
52. Pagabeleguem S, Gimonneau G, Seck MT, Vreysen MJB, Sall B, Rayaissé J-B, et al. A Molecular Method to Discriminate between Mass-Reared Sterile and Wild Tsetse Flies during Eradication Programmes That Have a Sterile Insect Technique Component. PLoS Negl Trop Dis (2016), 10(2): e0004491..
53. Rayaisse JB, Kröber T, McMullin A, Solano P, Mihok S, Guerin PM. Standardizing visual control devices for tsetse flies: West African species *Glossina tachinoides*, *G. palpalis gambiensis* and *G. morsitans submorsitans*. *PLoS Negl Trop Dis*. 2012;6(2):e1491. doi:10.1371/journal.pntd.0001491.
54. Rayaisse, Jean – Baptiste, Salou Ernest, Courtin Fabrice, Yoni Wilfrid, Barry Issiaka, Dofini Fabien, Kagbadouno Moise, Camara Mamadou, Torr Steve, Solano Philippe. Baited-boats: An innovative way to control riverine tsetse, vectors of sleeping sickness in West Africa. Parasites & Vectors (2015) 8:236.
55. Rayaisse JB, Courtin, F., Akoundjin, M., Cesar, J. et Solano, P. Influence of anthropisation on local vegetation and tsetse abundance in southern Burkina Faso. Parasite, 2009, *16*, 21-28
56. Rouamba J., Jamonneau Vincent, Sidibé I., Solano Philippe, Courtin Fabrice. Impact de la dynamique de peuplement sur la distribution des glossines et des trypanosomoses dans la boucle du Mouhoun (Burkina Faso). *Parasite - Journal de la Société Française de Parasitologie*, (2009), 16 (1), p. 11-19. ISSN 1252-607X.
57. Salou, E, RAYAISSÉ J.B., LAVEISSIÈRE C., SANON A. & SOLANO P. “Interactions comportementales et rythmes d'activité de *Glossina palpalis gambiensis* et G. tachinoides (Diptera : Glossinidae) en galerie forestière au Burkina Faso” [Behavioral interactions and rhythms of activity of *Glossina palpalis gambiensis* and *G. tachinoides* (Diptera: Glossinidae) in a forest gallery in Burkina Faso]. *Parasite (Paris, France)* vol. 19,3 (2012): 217-25. doi:10.1051/parasite/2012193217.
58. Salou E, Rayaisse JB, Kaba D, Djohan V, Yoni W, Barry I, Dofini F, Bouyer J, Solano P. Variations in attack behaviours between *Glossina palpalis gambiensis* and *G. tachinoides* in a gallery forest suggest host specificity. Med Vet Entomol. 2016 Dec;30(4):403-409. doi: 10.1111/mve.12187. Epub 2016 Aug 11. PMID: 27513602.
59. Solano P, Salou E, Rayaisse JB, Ravel S, Gimonneau G, Traore I, Bouyer J. Do tsetse flies only feed on blood? Infect Genet Evol. 2015 Dec;36:184-189.
