# Supplementary material for: An atlas to support the progressive control of tsetse-transmitted animal trypanosomosis in Burkina Faso
Source: Parasit Vectors. 2022 Mar 4;15:72. doi: 10.1186/s13071-021-05131-4 (PMC8895521; doi:10.1186/s13071-021-05131-4)
Supplement: Supplementary file 2 — Additional file 2: Text S2. Structure of the tsetse database [file 13071_2021_5131_MOESM2_ESM.docx]

**Text S. The structure of the database on tsetse and African animal trypanosomosis in Burkina Faso**

Additional file 2: Text_S2. Tsetse fly

Table “**Ento_Survey"**  (trap level)

1. Survey_Id. A unique numeric identifier of each record in the Table
2. Trap_Name. A unique alpha-numeric code from trap code modified
3. Trap_Name_Sheet. original code as recorded in field
4. Trap_Type. Type of trap.
5. Trap_Attr. ‘1’ identifies the trap where attractant was used.
6. Attr_Type. Odour attractant used in the traps
7. Lat. Latitude of the survey trap in decimal degrees (Datum: WGS84).
8. Long. Longitude of the survey trap in decimal degrees (Datum: WGS84).
9. Alt. Altitude of the survey trap in meter.
10. Date_St. Starting date (day/month/year) of the trapped survey.
11. Time_St. Starting hour of the trapped survey
12. Date_En. Ending date (day/month/year) of the trapped survey.
13. Time_En. Ending hour of the trapped survey
14. Trap_Time. Duration of trapping (in days).
15. Location_Id. A foreign identifier of location
16. Source_Id. A foreign identifier of data source
17. Epecies_No. Number of species of tsetse captured by the relevant trap.
18. TseTse_Interv. ‘1’ identifies the existence of interventions against tsetse
19. TseTse_Interv_Note. It reports what interventions against tsetse were ongoing in the study area at the time of the survey.

Table “**Trapped_Tsetse**» (trap level)

1. Tsetse_Id. A unique numeric identifier of each record in the Table
2. Species. Name of species or subspecies of tsetse fly.
3. M_No. Total number of male caught
4. F_No. Total number of female caught
5. ND_No. Total number of none determined sex caught
6. Flies_No. Total number of flies caught.
7. Survey_Id. A foreign numeric identifier of survey trap.

Table “**Ento_Survey_Lit"** (site level)

1. Survey_Id_Lit. A unique numeric identifier of each record in the Table
2. Trap_Type. Type of trap used for data collection.
3. Trap_Attr. ‘1’ identifies the trap where attractant was used.
4. Attr_Type. Odour attractant used for capture
5. Month_St. Starting month of the survey.
6. Month_En. Ending month of the survey.
7. Year_St. Starting year of the survey.
8. Year_En. Ending month of the survey.
9. Trap_Time. Duration of trapping (in days).
10. Trap_No. Total number of traps deployed in the survey site.
11. Month_No. Total number of month of survey.
12. Absence_Presence. Flies absence or presence (Presence : 1, Absence : 0)
13. Location_Id. foreign numeric identifier of location
14. Source_Id. A foreign numeric identifier of data source
15. TsetTse_Interv. ‘1’ identifies the existence of interventions against tsetse.
16. TseTse_Interv_Note. It reports what interventions against tsetse were ongoing in the study area at the time of the survey.

Table “**Trapped_Tsetse_lit**» (site level)

1. Tsetse_Id_Lit. A unique numeric identifier of each record in the Table
2. Species. Name of the species or subspecies of tsetse fly.
3. M_No. Total number of male caught
4. F_No. Total number of female caught
5. Flies_No. Total number of flies caught.
6. Flies_AD. Flies apparent density (flies/trap/day).
7. Survey_Id_Lit. A foreign numeric identifier of Survey

The table **“Source of data”**

1. Source_id. A unique numeric identifier of each record in the Table
2. Institution. Name of the national institution or project that generate the data source
3. Initials. Initials of the first author surname of the input file or scientific paper. Where there is more than one initial, they are separated with a space, e.g. J B. For institution, the thirst letter of the abbreviation of its name were used as initials (e.g. C for CIRDES).
4. Author. Name of the first author of the input file or scientific paper.
5. All_Authors. Names of all authors of the input file or scientific paper.
6. Title. Title of the paper
7. Year. Year of publication of the paper or survey for unpublished data
8. File_Name. Standardized naming. For PDF files, it include author(s) name(s), year of publication (e.g. Rayaisse_et_al_2010.pdf). For unpublished paper, it include activity, starting month, ending month and survey year of activity (e.g. Evaluation de barriere entomologique_juillet_novembre_2013.doc).
9. AaT_Data. ‘1’ identifies documents containing spatially referenced data on AAT.
10. Tsetse_Data. ‘1’ identifies documents containing spatially referenced data on tsetse absence presence and/or abundance.
11. Tsetse_Infect_Data. ‘1’ identifies documents containing spatially referenced data on tsetse infection.
12. Published. ‘1’ identifies scientific papers, whereas ‘0’ refers to any other document.
13. Raw_Data. ‘1’ identifies documents for which raw data are available,
14. Publisher. Name of the publisher of the journal.
15. Journal. Name of the journal where the paper is published
16. Source_Note. Field containing relevant comments concerning the source.

Table “**Geo_Data"**

1. Location_Id. A unique numeric identifier of each location in the table
2. Admin_1. Name of the first subnational administrative unit (*région*) where the site is located (as reported in the input file)
3. Admin_2. Name of the second subnational administrative unit (*Province*) where the site is located (as reported in the input file)
4. Admin_3. Name of the third subnational administrative unit (*department*) where the site is located (as reported in the input file).
5. Lat. Latitude of the study site or village in decimal degrees (Datum: WGS84).
6. Long. Longitude of the study site or village in decimal degrees (Datum: WGS84). LAT/LONG coordinates are specific to the site listed in LOCATION_NAME.
7. Location_Note. relevant comments concerning the location.
