# Supplementary material for: An atlas to support the progressive control of tsetse-transmitted animal trypanosomosis in Burkina Faso
Source: Parasit Vectors. 2022 Mar 4;15:72. doi: 10.1186/s13071-021-05131-4 (PMC8895521; doi:10.1186/s13071-021-05131-4)
Supplement: Supplementary file 3 — Additional file 3: Text S3. Structure of the African animal trypanosomosis database [file 13071_2021_5131_MOESM3_ESM.docx]

## Additional file 3: Text_S3. African animal trypanosomosis

Table “**Epi_data_raw » for epidemiological** (animal level)

1. Epi_Id. A unique numeric identifier of each animal in the table
2. Anim_Code. A code identifier of each animal recorded from field.
3. Location_Id. A foreign numeric identifier of location where the survey was carried out.
4. An_Spec_Id. A foreign numeric identifier of animal species examined (i.e. 1: Bovine).
5. Sex. Sex of animal
6. Age. Age of animal.
7. Unit. The unit of age (i.e. month, year).
8. Husb_An. Animal husbandry system (prevalently sedentary)
9. Date. Date of surevey
10. An_Status. The status of individual animal
11. PCV. . Average Packed-Cell-Volume for individual animal.
12. *Tc*. *T. congolense* (presence 1 or absence : 0)
13. *Tcs*. *T. congolense* *savanah* (presence: 1 or absence : 0)
14. *Tcf*. *T. congolense* *forest* (presence: 1 or absence: 0).
15. *Tck*. *T. congolense kilifi* (presence: 1 or absence : 0).
16. *Tct*. *T. congolense tsavo* (presence: 1 or absence : 0)
17. *Tv*. *Trypanosoma vivax* (presence: 1 or absence : 0).
18. *Tb. Trypanosoma brucei s.l.* (presence: 1 or absence : 0)
19. *Tbb* *Trypanosoma brucei brucei* (presence: 1 or absence: 0).
20. *Tbg. Trypanosoma brucei gambiense (*presence: 1 or absence : 0).
21. *Tsi. T. simia (*presence: 1 or absence : 0).
22. Other_Infect. Multiple infections (mixed, triple) of individual animal (i.e. *T.c* +*T.v*, *T.c* + *T.b*, *T.c*+*T.v*+*T.si*)
23. T. Number of trypanosoma species diagnosed on one individual animal.
24. Random. The random approach used for animals and survey location selection (i.e. 1 : random)
25. Random_Note. It describes how a random approach was used (e.g. in a study designed to assess the general epidemiological situation in an area), or a purposeful one (e.g. where specific villages/areas/herds/animals are investigated because of some peculiar features of theirs).
26. Longitudinal. It describes whether data were from a longitudinal study
27. TseTse_Interv. . ‘1’ identifies the existence of interventions against tsetse.
28. TseTse_Interv_Note. It reports what interventions against tsetse were ongoing in the study area at the time of the survey, or in the recent past prior to the survey.
29. Chemoth. It includes information related to the use of therapeutic and/or prophylactic antitrypanosomal drugs (1. Yes/0. No).
30. Diagn_Id. A foreign numeric identifier of a diagnostic method used.
31. Source_Id. A foreign numeric identifier of the source of data from the table “source”.

Table “**Epi_data_lit »** (herd level)

1. Epi_data_Litt_Id. A unique numeric identifier of each record in the Table.
2. Location_Id. A foreign numeric identifier of location where the survey was carried out
3. Source_Id. A oreign numeric identifier of data source
4. Month_St. Starting month of the survey.
5. Month_En. Ending month of the survey.
6. Year_St. Starting year of the survey.
7. Year_En. Ending year of the survey.
8. Sample_Size. Total number of animals sampled.
9. An_Spec_Id. A foreign numeric identifier of animal species examined (i.e. 1: Bovine).
10. Sex. Sex of animals.
11. Age. Age of animals.
12. Husb_Syst. Animal husbandry system (prevalently sedentary).
13. PCV. Average Packed-Cell-Volume for the survey herd.
14. Tc. Total number of animals positive to the test for *T. congolense* or
15. Tcs. Total number of animals positive to the test for *T. congolense* or
16. Tcf. Total number of animals positive to the test for *T. congolense* or
17. Tck. Total number of animals positive to the test for *T. congolense* or
18. Tct. Total number of animals positive to the test for *T. congolense* or
19. Tv. Total number of animals positive to the test for *Trypanosoma vivax* or
20. Tb. Total number of animals positive to the test for *T. brucei s.l* or
21. Tbb. Total number of animals positive to the test for *T. brucei s.l* or
22. Tbg. Total number of animals positive to the test for *T. brucei s.l* or
23. Tsi. Total number of animals positive to the test for *T. simiae* or
24. Other_Infect. Multiple infections (mixed, triple) of individual animal (i.e. *T.c* +*T.v*, *T.c* + *T.b*, *T.c*+*T.v*+*T.si*)
25. T. Number of animals positive to the test for any of all the species of trypanosomes under study
26. Aat_Presence. ‘Yes’ if the African animal trypanosomosis is present, “No” if the disease is not present. This field might be useful when the input file only reported the absence or presence of AAT, but it reported neither the number of infections, not the prevalence.
27. Tc_Pr. *T.c* prevalence
28. Tcs_Pr. *T.cs* prevalence.
29. Tcf_Pr. *T.cf* prevalence.
30. Tck_Pr. *T.ck* prevalence.
31. Tct_Pr. *T.ct* prevalence
32. Tv_Pr. *T.v* prevalence
33. Tb_Pr. *T.b* prevalence
34. Tbb_Pr. *T.bb* prevalence
35. Tbg_Pr. *T.bg* prevalence
36. Tsi_Pr. *T.si* prevalence.
37. Other_Infect_Pr. Multiple infection prevalence.
38. T_Pr. AAT prevalence.
39. TseTse_Interv. ‘1’ identifies the existence of interventions against tsetse.
40. TseTse_Interv_Note. Reports which interventions against tsetse were ongoing in the study area at the time of the survey.
41. Chemoth. It includes information related to the use of therapeutic and/or prophylactic antitrypanosomal drugs.
42. Chemoth_Date. Recent date of the use of therapeutic and/or prophylactic antitrypanosomal drugs for animals herd treatment.
43. Chemoth_Note. relevant comments concerning last treatment of animals
44. Random. “1” if the approach used for animals and survey location selection is randomized
45. Describes whether a random approach was used (e.g. in a study designed to assess the general epidemiological situation in an area), or a purposeful one (e.g. where specific villages/areas/herds/animals are investigated because of some peculiar features of theirs).
46. Diagn_Id. A foreign numeric identifier of the diagnostic method used.
47. Longitudinal. Describes whether data were extracted from a longitudinal study.

Table “**Chemotherapy»**

1. Chemoth_Id. A unique numeric identifier of each report.
2. Chemoth_Date. Date of use of therapeutic and/or prophylactic antitrypanosomal drugs
3. Chemoth_Cause. Cause of treatment
4. Prod_Used. The drug used
5. Dose. The dose of drug used
6. Who_Treat. The person who treat the animal
7. Epi_Id. A foreign numeric identifier of individual animal from table “Epi_Data_Raw.

Table “**Diagnostic_Method»**

1. Diagn_id. A unique numeric identifier for each diagnostic method.
2. Diagn_Meth. Diagnostic method used for trypanosome identification (i.e. Buffy coat technique, PCR)
3. Note. relevant comments concerning the diagnostic method.

Table “**Animal_Species»**

1. An_Spec_Id. A unique numeric identifier for each animal species (i.e. 1 for Bovine or Ovine).
2. Species. Species of animal
3. Breed. Animal breed (e.g. Zebu or *Taurine)*
4. Note. relevant comments concerning on animals species and breed of sample.

The tables Data source and geographic have the same structures for the two database.

The table **“Source of data”**

1. Source_id. A unique numeric identifier of each record in the Table
2. Institution. Name of the national institution or project that generate the data source
3. Initials. Initials of the first author surname of the input file or scientific paper. Where there is more than one initial, they are separated with a space, e.g. J B. For institution, the thirst letter of the abbreviation of its name were used as initials (e.g. C for CIRDES).
4. Author. Name of the first author of the input file or scientific paper.
5. All_Authors. Names of all authors of the input file or scientific paper.
6. Title. Title of the paper
7. Year. Year of publication of the paper or survey for unpublished data
8. File_Name. Standardized naming. For PDF files, it include author(s) name(s), year of publication (e.g. Rayaisse_et_al_2010.pdf). For unpublished paper, it include activity, starting month, ending month and survey year of activity (e.g. Evaluation de barriere entomologique_juillet_novembre_2013.doc).
9. AaT_Data. ‘1’ identifies documents containing spatially referenced data on AAT.
10. Tsetse_Data. ‘1’ identifies documents containing spatially referenced data on tsetse absence presence and/or abundance.
11. Tsetse_Infect_Data. ‘1’ identifies documents containing spatially referenced data on tsetse infection.
12. Published. ‘1’ identifies scientific papers, whereas ‘0’ refers to any other document.
13. Raw_Data. ‘1’ identifies documents for which raw data are available,
14. Publisher. Name of the publisher of the journal.
15. Journal. Name of the journal where the paper is published
16. Source_Note. Field containing relevant comments concerning the source.

Table “**Geo_Data"**

1. Location_Id. A unique numeric identifier of each location in the table
2. Admin_1. Name of the first subnational administrative unit (*région*) where the site is located (as reported in the input file)
3. Admin_2. Name of the second subnational administrative unit (*Province*) where the site is located (as reported in the input file)
4. Admin_3. Name of the third subnational administrative unit (*department*) where the site is located (as reported in the input file).
5. Lat. Latitude of the study site or village in decimal degrees (Datum: WGS84).
6. Long. Longitude of the study site or village in decimal degrees (Datum: WGS84). LAT/LONG coordinates are specific to the site listed in LOCATION_NAME.
7. Location_Note. relevant comments concerning the location.
